# Toxic Shock Syndrome Toxin-1-Mediated Toxicity Inhibited by Neutralizing Antibodies Late in the Course of Continual *in Vivo* and *in Vitro* Exposure

**DOI:** 10.3390/toxins6061724

**Published:** 2014-05-30

**Authors:** Norbert Stich, Nina Model, Aysen Samstag, Corina S. Gruener, Hermann M. Wolf, Martha M. Eibl

**Affiliations:** 1 Biomedizinische ForschungsgmbH Lazarettgasse 19/2, Vienna A-1090, Austria; E-Mails: Norbert.Stich@biomed-research.at (N.S.); Nina.Model@biomed-research.at (N.M.); Corina.Gruener@biomed-research.at (C.S.G.); 2 Immunology Outpatient Clinic, Schwarzspanierstrasse 15, Vienna A-1090, Austria; E-Mails: office@itk.at (A.S.); hermann.wolf@itk.at (H.M.W.)

**Keywords:** toxic shock syndrome toxin-1, TNF-alpha, IL-2, neutralizing antibody, rabbit animal model, postexposure treatment, continual stimulation

## Abstract

Toxic shock syndrome (TSS) results from the host’s overwhelming inflammatory response and cytokine storm mainly due to superantigens (SAgs). There is no effective specific therapy. Application of immunoglobulins has been shown to improve the outcome of the disease and to neutralize SAgs both *in vivo* and *in vitro*. However, in most experiments that have been performed, antiserum was either pre-incubated with SAg, or both were applied simultaneously. To mirror more closely the clinical situation, we applied a multiple dose (over five days) lethal challenge in a rabbit model. Treatment with toxic shock syndrome toxin 1 (TSST-1) neutralizing antibody was fully protective, even when administered late in the course of the challenge. Kinetic studies on the effect of superantigen toxins are scarce. We performed *in vitro* kinetic studies by neutralizing the toxin with antibodies at well-defined time points. T-cell activation was determined by assessing T-cell proliferation (3H-thymidine incorporation), determination of IL-2 release in the cell supernatant (ELISA), and IL-2 gene activation (real-time PCR (RT-PCR)). Here we show that T-cell activation occurs continuously. The application of TSST-1 neutralizing antiserum reduced IL-2 and TNFα release into the cell supernatant, even if added at later time points. Interference with the prolonged stimulation of proinflammatory cytokines is likely to be *in vivo* relevant, as postexposure treatment protected rabbits against the multiple dose lethal SAg challenge. Our results shed new light on the treatment of TSS by specific antibodies even at late stages of exposure.

## 1. Introduction

Sepsis is the most common cause of death in critically ill patients, and it results from the overwhelming inflammatory response of the host [1], as well as from the inability of the immune system to limit bacterial spread during an ongoing infection. Numerous clinical trials have been conducted with the aim of blocking the uncontrolled inflammatory cascade, but with limited success [2,3,4,5,6,7]. The most common causative organisms in patients with sepsis are *Staphylococcus aureus*, *Pseudomonas aeruginosa* and *Escherichia coli* [8]. Whereas in the 1980s the most frequently identified organisms were Gram-negative bacteria, Gram-positive bacteria have accounted for the greatest proportion of hospital admissions with sepsis in the last decade [8,9]. This might be a consequence of the increasing prevalence of multiresistant organisms such as methicillin-resistant *S. aureus* [10] and the wider use of prostheses and invasive vascular devices [11]. *S. aureus* causes significant illnesses, including pneumonia, acute kidney injury, infective endocarditis, and toxic shock syndrome (TSS) [12]. Major contributors to these diseases are superantigens (SAgs), such as toxic shock syndrome toxin- 1 (TSST-1) and staphylococcal enterotoxin B (SEB), both of which remarkably hyperactivate the host’s inflammatory response.

Numerous efforts have been undertaken to develop a specific therapy for TSS [13,14]. Therapies of sepsis have included the application of intravenous immunoglobulin (IVIG), which has been only partially useful [15]. Hyperimmune IVIG could be produced by vaccination with a recombinant attenuated SAg vaccine. These immunoglobulins could offer the advantage of both neutralizing SAgs and modulating the inflammatory reaction, e.g., by lowering the levels of circulating cytokines [16,17,18]. Since there is a strong relation between toxicity and increased serum levels of cytokines, many therapeutic approaches in animal models aimed at blocking these proinflammatory mediators [19,20,21]. However, anticytokine treatments have not been successful in clinical trials since sepsis is a complex process involving excessive and suppressed inflammatory and immune responses [22].

In studies using staphylococcal enterotoxin B (SEB), it has been shown that mouse and non-human primates were protected from SEB-induced TSS by the use of antibodies up to 4 h after toxin exposure [23]. Larkin *et al*. investigated the effect of monoclonal Fab fragments and whole monoclonal antibodies against SEB in an extensive kinetic study. Some of these antibodies bound their targets with very high affinity, and the protective effect in the mouse toxic shock model reached 68% [24]. In a rabbit model, IVIG reduced the toxic effects of exotoxins, but mainly when immunoglobulins and toxins were injected simultaneously and not when the application of antiserum was delayed [25]. Similar results were obtained *in vitro* when human PBMCs were stimulated with SEB, and T-cell responses could be inhibited by antibodies up to 12 h after SAg exposure [24].

In a rabbit infection model of TSS using *S. aureus* producing TSST-1, fatal disease could be inhibited by application of TSST-1-neutralizing monoclonal antibodies [26]. Notably, for protection in this model, the antibodies had to be given constantly before and during the challenge (on days−1, 0, 1). We chose the rabbit model, since the sensitivity and the susceptibility of humans and rabbits to SAgs is comparable. Furthermore, the pathological effects of SAgs are highly similar in humans and rabbits [12,27,28,29]. In the latter publication it was shown that rabbits could be protected from lethal pneumonia after having been challenged with SAg (SEB) followed by delayed administration of IVIG (up to 48 h). Moreover, it was shown that rabbit immune serum was protective when given prior to challenge.

Another strategy to limit the overproduction of cytokines and to elicit a powerful antibody response against SAgs such as TSST-1 is achieved by vaccination. Rabbits which received TSST-1 toxoids developed strong antibody titers that neutralized TSST-1 in TSS models *in vitro* and *in vivo* [30]. Mice vaccinated with mutant TSST-1 could be protected against *S. aureus*-induced septic death by neutralizing antibodies and downregulation of IFNγ production [16]. Anti-SAg antibodies are widespread among the human population, and there is a good correlation between antibody titers and the inhibition of superantigenic effects of these toxins [31]. SAg-specific antibodies from pooled sera could suppress T-cell proliferation *in vitro* and protect mice against SAg-induced TSS [31]. In these previous studies, the toxin-neutralizing effect of antibodies was mostly analyzed by *in vivo* and *in vitro* systems in which antibodies were present before toxin challenge (e.g., through vaccination): Antibodies and toxins were applied either simultaneously or after pre-incubation, or antibodies were given after a single challenge with toxin. However, patients usually receive clinical treatment several hours (if not days or weeks) after exposure to pathogens and their toxins, and during this lag period they are continuously exposed to an ongoing production of bacterial toxins. Continual exposure was achieved by inserting a pump, which showed that lethal doses were much lower under these conditions than with a bolus injection [27,30]. In the present study we applied defined amounts of recombinant TSST-1 wild-type (rTSST-1 wt) within a five-day period in a rabbit multiple dose challenge model, thus mimicking the clinical situation of systemic Gram-positive bacterial infection with continuous exposure to toxins over a longer time period. In this model, treatment with neutralizing antibodies given late in the course of toxin challenge fully protected from toxin-induced lethality.

A major component involved in the pathogenesis of Gram-positive sepsis is the ability of bacterial antigens to induce an exaggerated release of proinflammatory and immune cell-activating cytokines, e.g., TNFα, IFNγ, IL-6 and IL-2. The superantigenic characteristic to hyperactivate T cells might play a role in this context [12,32,33]. The effect of postexposure antibody treatment on T-cell activation, cytokine gene expression and the release of inflammatory cytokines following TSST-1 stimulation was assessed in an *in vitro* system. Toxin-neutralizing antiserum was added to PBMC cultures at different time points after superantigen exposure. Cytokine mRNA expression and protein secretion were analyzed in parallel in order to monitor gene expression patterns and cytokine release. Our results demonstrate that the release of cytokines into the supernatant decreases following neutralization of the stimulus, as long as the maximum cytokine concentration in the supernatant has not been reached. Cytokine mRNA is continuously produced upon stimulation with SAg toxin and is labile. Neutralization of the toxin stops cytokine gene transcription completely, if given within the first hours after toxin exposure.

## 2. Results and Discussion

### 2.1. Multiple Dose Lethal Challenge and Protection by Neutralizing Antibody

We first examined whether rabbits could be protected against multiple dose lethal challenge with rTSST-1 by pre-incubation of toxin with antiserum before application. The control group (two rabbits) received 30 µg of toxin per dose as explained in the legend to Table 1 (in this model the lethal dose range was between 20 and 40 µg of rTSST-1 wt: number of rabbits that survived/number of animals that were challenged: 20 µg per dose: 0/4; 30 µg/dose: 1/11; 40 µg per dose: 0/2; 100 µg per dose: 0/4). The rabbits died at days 5 and 6. The second group (three rabbits) received neutralized toxin (as described in the Experimental Section) for each injection. All rabbits survived until nine days *post* challenge. The third group (three rabbits) received toxin until day 3 (six injections) and neutralized toxin on days 4 and 5. All rabbits survived, were free of severe symptoms on day 5 and day 7, as determined by a veterinarian observing behavior, vital signs, mucosa of the nose, eyes and anus, and food and water uptake. The rabbits were euthanized 9 days *post* challenge.

We then examined whether rabbits could be protected against lethal challenge by passive transfer of TSST-1 antibodies injected at a site different from the one used for toxin administration. Multiple doses of 40 µg or 30 µg rTSST-1 were given (see legend to Table 1). One group of rabbits was injected with 2 mL of antiserum as described in the Experimental Section at a different site on days 2 and 3, while another group received 2 mL of antiserum on day 3 (after the 6th toxin dose), day 4 (after dose 7) and day 5 (after dose 9) (three doses). For timeline and detailed description of treatment see Experimental Section. The survival rates of all rabbits were monitored during these five days of continual challenge and for a further 7–9 days (Table 1).

**Table 1:** Survival of rabbits challenged with multiple doses rTSST-1 and effect of anti-TSST-1 antiserum.

Antiserum raised against	Days of treatment	Survival (No. of animals that survived/total No. of animals challenged)	
-	-	0/4 *	
Negative/irrelevant serum	1–5	0/3 ^++^	
TSST-1 variant	2, 3	5/5 **	*p* = 0.02 ***
TSST-1 variant	3–5	5/5 **	*p* = 0.02
TSST-1wt	3– 5	5/5 **	*p* = 0.02

Notes: * Of 4 rabbits, 2 rabbits received 2 doses of 30 µg rTSST-1 (days 1–4) and 1 dose of 30 µg rTSST-1 (day 5), and 2 rabbits received 2 doses of 40 µg rTSST-1 (days 1–4) and 1 dose of 40 µg (day 5); ^++^ Two rabbits were treated with antiserum raised against *S. aureus* alpha toxin, one rabbit was treated with pre-immune serum (days 1–5); ** Of 5 rabbits, 3 received 2 doses of 30 µg rTSST-1 (days 1–4) and 1 dose of 30 µg rTSST-1 (day 5), and 2 rabbits received 2 doses of 40 µg rTSST-1 (days 1–4) and 1 dose of 40 µg rTSST-1 (day 5); *** Statistical significance of the difference between treatment and control group determined by chi-square analysis.

In these experiments, rabbits survived the rTSST-1 challenge when treated with antiserum either on days 2 and 3, or on days 3, 4 and 5. Both types of antiserum (gained via immunization with TSST-1-wild-type or TSST-1-variant) were equally protective. Rabbits without protective antiserum died between four days and one week after the first challenge. Our data demonstrate the efficacy of anti-TSST-1 antibodies for the protection of rabbits, even when given delayed during continual exposure to bacterial toxin in an animal model that more closely resembles the clinical situation in patients suffering from systemic gram-positive bacterial infection.

Multiple dose lethal challenge continual exposure, during which toxin concentrations are accumulated (over several days of treatment), leads to death. Since rTSST-1 challenge is not lethal for three days, treatment with immunoglobulin at that time point may have a prophylactic effect by neutralizing and preventing further accumulation of the toxin.

### 2.2. Inhibition of T-Cell Proliferation

**Figure 1:**
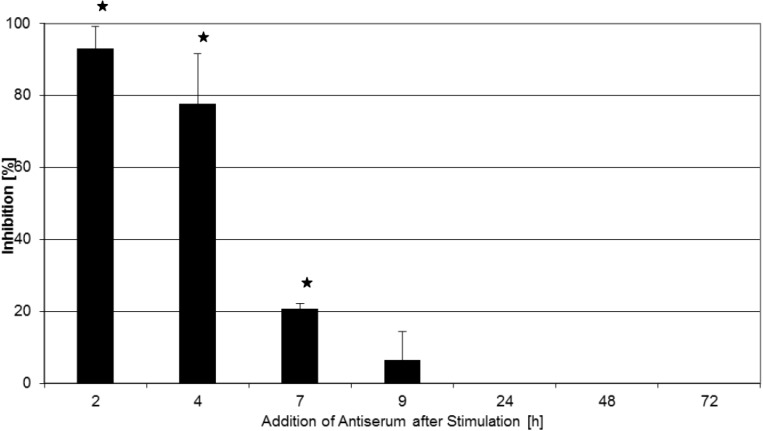
Inhibition of rTSST-1-induced T-cell proliferation by antiserum. Human PBMC were stimulated with 0.3 ng/mL rTSST-1 wt and cultured for four days as described in the Experimental Section. Antiserum generated against rTSST-1 was added at 2 h (n = 8), 4 h (n = 8), 7 h (n = 4), 9 h (n = 4), 24 h (n = 4), 2d (n = 4) or 3d (n = 4) after stimulation in a final dilution of 1:100. Each experiment was carried out in triplicate. Inhibition of proliferation is reported in percentage in relation to negative control serum, values represent the mean, and error bars indicate the standard deviation of multiple experiments. PBMC proliferative response following stimulation with rTSST-1 wt in the presence of negative serum was 60,186 ccpm ± 28,002 (mean ± SD, n = 4). The term “negative serum” refers to a serum derived from animals not immunized against TSST-1, *i.e.*, preimmune serum. PBMC proliferative response following stimulation with rTSST-1 wt without antiserum was 43,388 ccpm ± 6510 (mean ± SD, n = 4). Background proliferative response of cells cultured in medium alone was 1020 ccpm ± 462 (mean ± SD, n = 4). % inhibition was calculated as described in the Experimental Section using the following formula: % inhibition = 100 – ((ccpm ^3^H thymidin incorporation in the presence of antiserum/ccpm ^3^H thymidin incorporation in control cultures containing negative serum) × 100). An asterisk indicates a statistically significant (*p* < 0.05) inhibition (paired Student’s *t*-test).

As T-cell hyperactivation is a crucial step in the pathogenesis of Gram-positive systemic inflammatory syndrome, and given the positive effect of antiserum treatment late in the course of toxemia as described above, we examined whether the antisera used in our animal model of continual toxin exposure could also inhibit superantigen activation of human T cells *in vitro* when applied after toxin stimulation of the cells. As a first readout for T-cell activation, T-cell proliferation assays were performed. As described in the Experimental Section, human PBMCs were stimulated with rTSST-1 wt, and antiserum was added at different times thereafter. Figure 1 shows that T-cell proliferation was nearly completely inhibited when the antiserum was added at 2 h and 4 h after TSST-1 stimulation. Addition at 7 h resulted in 20% inhibition of T-cell proliferation. Application of antiserum at later times did not have any influence on the TSST-1-induced proliferation of T cells. In the course of these experiments, two different neutralizing antibodies (raised against rTSST-1 wt or rTSST-1 variant) were used, but the inhibitory effect obtained using antiserum against rTSST-1 variant (one of the mutations is at the T-cell receptor binding site) or rTSST-1 wt was comparable: ^3^H thymidin incorporation at the end of the four-day culture period in the presence of negative serum was 60,186 ccpm ± 28,002 (mean ± SD, n = 4). When antiserum against rTSST-1 wt was added at 4 h, it was 25,110 ccpm (mean, n = 2). When antiserum raised against rTSST-1 mutant was added at 4 h, ^3^H thymidin incorporation was 19,783 ccpm (mean, n = 2). Therefore, results from both antisera were pooled for analysis.

These results suggest that shortly after stimulation of the cells, before certain activation steps, e.g., IL-2 induction and IL-2 release into the culture supernatant have reached a threshold, antisera can still significantly inhibit superantigen-induced T-cell activation. Thereafter, T-cell activation is self-perpetuating, and it cannot be inhibited, even by complete neutralization of the stimulus still present in the cell culture.

### 2.3. Kinetics of Inhibition of Cytokine Expression after rTSST-1 wt Stimulation for Defined Time Periods

There is a strong association between superantigen toxicity and an exaggerated release of proinflammatory and immune cell activating cytokines [19,20,21]. Several therapeutic approaches in animal models aimed at blocking the action of these proinflammatory mediators [23,24,25]. To examine the effect of postexposure immune prophylaxis by antiserum treatment on the levels of superantigen-induced cytokines, we applied an *in vitro* system for monitoring the kinetics of the cytokine expression profile in human PBMC following stimulation with recombinant rTSST-1 wild-type (rTSST-1 wt) and sequential neutralization of the toxin. We chose the expression of two cytokines produced early *in vitro* and also early after applying the toxin *in vivo*. We selected IL-2 as an indicator of T-cell activation and determined TNFα as one of the cytokines produced by T cells and non-T cells that play a pivotal role in superantigen-mediated toxicity. The appropriate concentration of rTSST-1 wt (1 ng/mL) had been chosen by previous titration experiments (data not shown).

**Figure 2:**
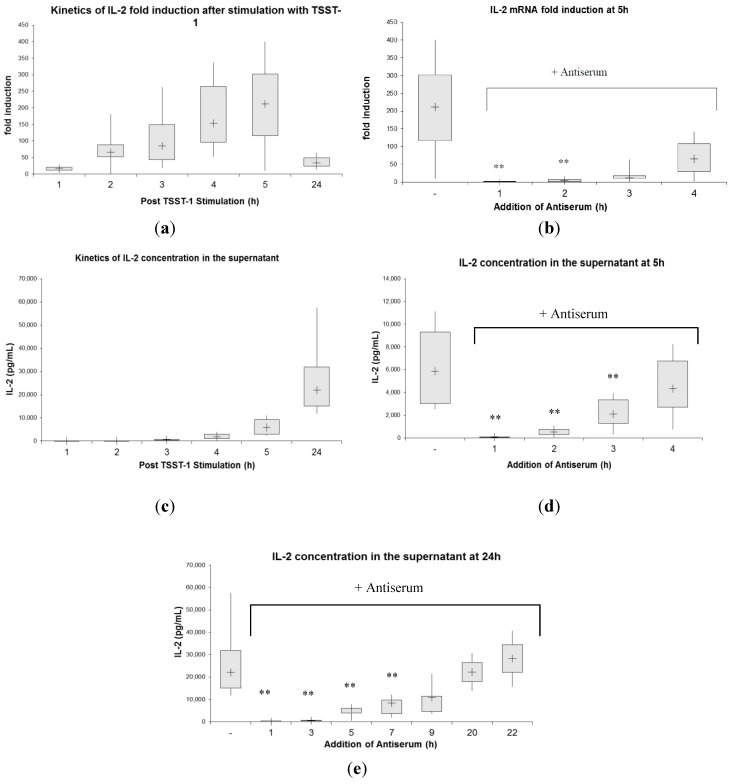
(**a**) Kinetics of IL-2 mRNA expression in human PBMC after stimulation with TSST-1 for designated time intervals. At the corresponding time point, cells were harvested for IL-2 mRNA fold induction analysis via RT PCR as indicated in the Experimental Section. The supernatant of these cultures served for determination of IL-2 concentration (see Figure 2c); (**b**) IL-2 gene activation in human PBMCs at 5 h after TSST-1 stimulation and inhibition at different time points with anti-TSST-1 antiserum. Antiserum was added at 1 h, 2 h, 3 h and 4 h followed by PBMC harvesting at 5 h. IL-2 mRNA expression was analyzed with real-time PCR as described in the Experimental Section. Stimulation with TSST-1 for 5 h without antiserum served as a control. Simultaneous addition of rTSST-1 wt and antiserum to PBMCs resulted in blocking of IL-2 mRNA expression (fold induction of 1; 1–2; median; interquartile range; n = 6). Simultaneous stimulation with rTSST-1 and negative serum led to a fold induction of 151, 109–167; (median, interquartile range) (n = 5); (**c**) IL-2 concentration in the supernatant of cultured human PBMCs stimulated with 1 ng/mL TSST-1 for indicated periods. At the designated time points, cells were harvested and IL-2 protein concentration was assessed by ELISA; (**d**) Amount of IL-2 after 5 h of stimulation with rTSST-1 wt and addition of antiserum at 1 h, 2 h, 3 h, and 4 h. When antiserum and TSST-1 were applied simultaneously for 5 h, we detected 367 pg/mL, 292-379 pg/mL; (median, interquartile range) IL-2 in the supernatant (n = 3) at this time point. PBMCs in medium alone for 5 h secreted 6 pg/mL IL-2, 0–43 pg/mL; (median, interquartile range) (n = 6). When negative serum and rTSST-1 were given simultaneously for 5 h, 3655 pg/mL, 2677–3944 (median, interquartile range) of IL-2 was detected in the supernatant (n = 3); (**e**) IL-2 secretion 24 h after stimulation. Antiserum was added to the culture at 1 h, 3 h, 5 h, 7 h, 9 h, 20 h, and 22 h and IL-2 secretion was determined at 24 h. The data presented are representative of 6 separate experiments. PBMCs in medium alone for 24 h secreted 27 pg/mL, 15–282 (median, interquartile range) IL-2 (n = 6). ** Statistically significant difference compared with stimulated PBMCs without antiserum using the Wilcoxon signed-ranks test (*p* < 0.001; n = 6). Box plot diagrams indicate the median (+), interquartile range (box) and minimum and maximum values (whiskers).

First, we examined human T-cell cytokine induction after rTSST-1 wt stimulation by studying the kinetics of IL-2 mRNA production applying RT PCR (Figure 2a,b). rTSST-1 wt led to a strong induction of IL-2 mRNA at 2 h, 3 h, 4 h, and 5 h with the maximum induction at 5 h (more than 200-fold) (Figure 2a). At 24 h, IL-2 mRNA production returned close to baseline. We then studied the kinetics of toxin neutralization by adding neutralizing antibody to the culture of toxin-stimulated PBMC at 4 time points (1, 2, 3 and 4 h after addition of TSST-1), and analyzed IL-2 mRNA expression after 5 h of stimulation (Figure 2b). Induction of IL-2 mRNA at 5 h was completely blocked when antiserum was added after 1 h and 2 h of TSST-1 stimulation. Addition of antiserum after 3 h or later was too late to completely inhibit induction of IL-2 message, but even if given as late as 4 h, it had a slight inhibitory effect on cytokine induction.

Moreover, the data depicted in Figure 2 indicate that the amount of superantigen-induced cytokine mRNA present in the cell culture results from a balance between ongoing production and continuous degradation, and that antiserum can significantly affect this steady state equilibration. Without antiserum, IL-2 mRNA levels were continuously increasing during five hours after toxin stimulation (Figure 2a). Neutralizing the toxin at 2, 3 and 4 h after toxin challenge led to a stop in newly produced IL-2 mRNA, with already induced cytokine mRNA being degraded. This process effectively led to decreased IL-2 mRNA levels when analyzed at 5 h (Figure 2b). Therefore, it appears likely that after neutralization of the stimulus, subsequent production of mRNA is abrogated and IL-2 mRNA decay seems to initiate immediately afterwards. The initiation of this effect could be observed immediately after neutralization. After 4 h of stimulation, newly produced mRNA accounts for more than 50% of the total IL-2 mRNA in the 5 h culture, and this increase is abolished by neutralization of the stimulus.

Studying the kinetics of IL-2 secretion after rTSST-1 wt exposure, we found that the highest concentration of IL-2 in our system was detected at 24 h in the supernatant of the cultures (22,043 pg/mL) (Figure 2c). We then examined whether IL-2 release could be inhibited by neutralizing TSST-1 antiserum if given as postexposure immune prophylaxis at defined time points. In the first set of experiments, IL-2 protein was analyzed in the supernatant after 5 h (Figure 2d). Subsequently, we followed IL-2 secretion over a period of 24 h, and added antiserum at 1 h, 3 h, 5 h, 7 h, 9 h, 20 h and 22 h (Figure 2e). The results depicted in Figure 2d,e show that early application of antiserum could efficiently inhibit the release of IL-2. An addition of antiserum at 1 h postexposure led to immediate and complete abrogation of IL-2 release. IL-2 concentration present at the respective time points of SAg neutralization did not significantly increase during the further culture period. Addition of neutralizing antibodies stopped protein secretion within the next hour. By comparing Figure 2c,d, one can see that protein levels with antiserum added at, e.g., 2 h (Figure 2d, IL-2 conc. 509 pg/mL), did not significantly exceed the levels produced in cultures at 3 h without antiserum (Figure 2c, IL-2 conc. 631 pg/mL).

However, even at late time points, such as neutralization after 7 h, a statistically significant inhibition could be observed, and the addition of antiserum at 20 h still showed a tendency towards reduced IL-2 secretion.

In summary, T-cell activation in human PBMCs occurred quickly after stimulation with rTSST-1 wt (as measured by IL-2 mRNA induction and secretion) and could be inhibited by postexposure antibody treatment at both, early (complete inhibition is given at 1 and 2 h after stimulation) and late, (partial inhibition) time points. After inhibition by antiserum, T-cell activation stopped immediately and IL-2 mRNA already induced was continuously degraded. On a protein level, IL-2 secretion continued slightly after inhibition through antiserum for a short period of time (~1 h), and remained at this level in the 5 h culture period.

**Figure 3:**
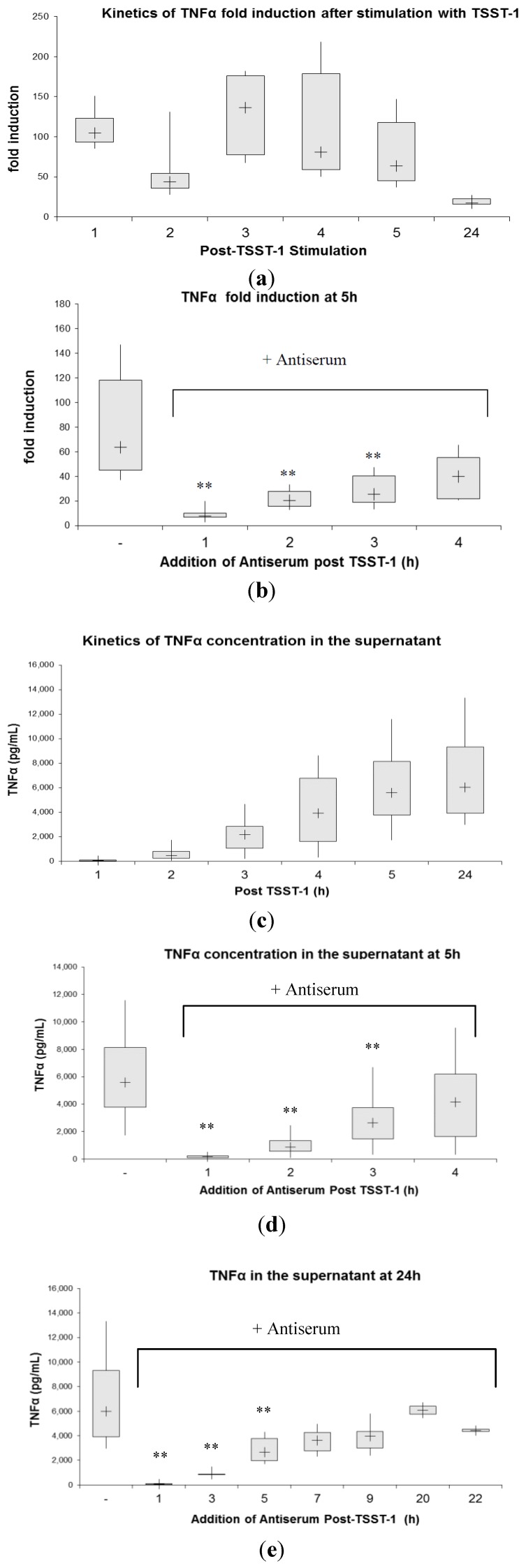
(**a**) Kinetics of TNFα mRNA expression in human PBMC after stimulation with TSST-1 for indicated periods. Cells were harvested and TNFα mRNA was analyzed via real-time PCR. The supernatant of these cultures served for analysis of TNFα protein secretion (see Figure 3c); (**b**) TNFα gene activation at 5 h after TSST-1 stimulation. Antiserum was added to PBMCs at 1 h, 2 h, 3 h, and 4 h and TNFα mRNA expression was analyzed at 5 h. Simultaneous addition of TSST-1 and antiserum to PBMCs resulted in blocking of TNFα mRNA expression (fold induction of 1.5; 1–2.5, median, interquartile range; n = 6). Simultaneous addition of rTSST-1 and negative serum led to TNFα fold induction of 4 5, 38–50 (median, interquartile range) (n = 6); (**c**) TNFα concentration in the supernatant of cultured human PBMCs stimulated with 1 ng/mL TSST-1 for indicated periods. TNFα concentration in the supernatant at 5 h (**d**) and 24 h (**e**) and the effect of antiserum. When antiserum and TSST-1 were applied simultaneously for 5 h, we detected 2 pg/mL, 1–13 (median, interquartile range) of TNFα in the supernatant (n = 3) at this time point. Negative serum applied simultaneously with rTSST-1 did not influence the accumulation of TNFα in the supernatant compared to rTSST-1 alone (4890 pg/mL, 4869–4911; median, interquartile range) (n = 3). PBMCs in medium alone secreted 0.5 pg/mL, 0–4; median, interquartile range TNFα at 5 h (n = 6) and 26 pg/mL, 15–74 (median, interquartile range) TNFα at 24 h (n = 6). ** Statistically significant difference compared with stimulated PBMCs without antiserum using the Wilcoxon signed-ranks test (*p* < 0,001; n = 6). Box plot diagrams indicate the median (+), interquartile range (box) and minimum and maximum values (whiskers).

Induction of the inflammatory cytokine TNFα followed different kinetics (Figure 3a). When TNFα mRNA induction was examined over a period of 24 h post rTSST-1 wt stimulation, TNFα mRNA was rapidly induced at 1 h to maximum levels remaining relatively constant up to 5 h. At 24 h, induction of TNFα mRNA was largely over. Our results indicate that TNFα was induced more rapidly than IL2 and maximum levels were reached as early as one hour after stimulation. Even in view of the different kinetics of mRNA-induction, addition of antiserum to the culture after stimulation could inhibit TNFα mRNA induction, although in contrast to IL-2, mRNA inhibition was not complete when antibody was added early (one hour) after stimulation (Figure 3b). Levels of TNFα mRNA produced were significantly reduced as antiserum inhibited newly induced mRNA, while mRNA already present was gradually degraded.

With respect to protein secretion, maximum levels of TNFα in the cell supernatant were detected at 24 h (Figure 3c) (6008 pg/mL). At an early time point (*i.e.*, 1 h), there was already a slight but statistically significant increase in TNFα protein release detectable. Addition of antiserum hampered protein synthesis at early time points, but also at later times (e.g., 5 h), though TNFα inhibition was less efficient than the one observed at IL-2 (Figure 3d,e). More than 9 h after stimulation, no inhibitory effect was observed.

## 3. Experimental Section

### 3.1. *In Vitro* Cytokine Gene Expression Assay

Peripheral blood mononuclear cells (PBMC) were isolated from heparinized blood of healthy human adults using density gradient centrifugation with Lymphoprep™ (Axis-Shield PoC, Oslo, Norway) as previously described [34]. PBMC were cultured in complete medium (RPMI 1640 medium (Gibco), 10% FCS (HyClone, Logan, UK), 2 mM L-glutamine (Invitrogen, Paisley, UK), 100 U/mL penicillin, and 100 µg/mL streptomycin (Invitrogen)) at a concentration of 5 × 10^6^/mL in 24-well flat-bottom tissue culture plates (Sarstedt, Newton, NC, USA) and stimulated with a final concentration of 1 ng/mL recombinant TSST-1 wildtyp (rTSST-1 wt) in humidified atmosphere (37 °C, 5% CO_2_) for time periods indicated in the text. Antiserum was added to the *in vitro*-stimulated PBMC at final dilutions of 1:100 at the time points indicated in the text.

At the end of stimulation, cells were resuspended in culture medium and transferred to Eppendorf tubes followed by centrifugation at 4 °C and 2000 × *g* for 5 min. Cell pellets were frozen at −20 °C before extracting RNA. The supernatants were saved for analysis of protein concentration via ELISA.

### 3.2. RNA Isolation and Reverse Transcription

RNA was extracted from frozen PBMC pellets using the *High pure RNA Isolation kit* from Roche. RNA was then reversely transcribed into cDNA using the *2xRT Kit* from Invitrogen (Paisley, UK) following the protocol of the manufacturer.

### 3.3. Primer Design

Gene-specific oligonucleotide primers were designed by hand and by primer design Primer Express^®^ v2.0 software from Applied Biosystems (Foster City, CA, USA). Primer pairs were synthesized at MWG/Eurofins Biotech (Heidelberg, Germany) and at Invitrogen. cDNA standards were prepared as previously described [34]. The primer sequences used for amplification of human cytokine cDNAs by real-time PCR were as follows:

IL-2-Forward:5'- AAACCTCTGGAGGAAGTG-3';IL-2-Reverse:5'- GTTCAGAAATTCTACAATGG-3';TNFα-Forward:5'- CTGTACCTCATCTACTCCC-3';TNFα-Reverse:5'- GAGAGGAGGTTGACCTTG-3';HPRT-Forward:5'- AGGCCATCACATTGTAGCCC-3';HPRT-Reverse:5'- GTTGAGAGATCATCTCCACCG-3'.

### 3.4. Quantitative Real-Time PCR (QRT-PCR) and Quantification

QRT-PCR was previously described [34]. In short, cDNA from samples and standards were simultaneously amplified on the same plate (MicroAmp, Applied Biosystems, Vienna, Austria) using an ABI Prism 7500-FAST (Applied Biosystems, Vienna, Austria) with the KAPA SYBR FAST Super Mix from PEQLAB (Erlangen, Germany) and ROX as reference dye. At the end of the amplification, a melting curve analysis was performed. The number of target cDNA copy numbers in the cellular samples was calculated by creating a standard curve where the cycles at threshold (CT) were plotted against the logarithmic values of the cDNA standard copy number. The housekeeping gene HPRT served as an internal standard. Fold induction of mRNA expression was assessed from values normalized for the expression of HPRT and then related to the mean values derived from unstimulated PBMCs of three human donors.

### 3.5. Lymphocyte Proliferation Assay

PBMC were isolated as described elsewhere [34]. In 96-well round-bottom tissue culture plates (Sarstedt, Newton, NC, USA), 1 × 10^5^ cells/well were then cultured in complete medium consisting of RPMI 1640 medium (Gibco), 10% FCS (HyClone, Logan, UK), 2 mM L-glutamine (Invitrogen, Paisley, UK), 100 U/mL penicillin, and 100 µg/mL streptomycin (Invitrogen). PBMC were stimulated in triplicate with rTSST-1 wt in final concentrations of 0.3 ng/mL. Sera of rabbits, immunized with rTSST-1 wt or rTSST-1 variant, were added at different times in a final dilution of 1:100. As a positive control, phytohaemagglutinin (PHA, Sigma–Aldrich, St. Louis, MO, USA) was used in a final dilution of 1:160. As a negative control, cells were grown in culture medium alone. Stimulated cells were cultured for four days in a humidified atmosphere (37 °C, 5% CO_2_). On day 3, 0.5 µCi/well 3H-thymidine (GE Healthcare, Chalfont St Giles, UK) was added and 18 h later plates were frozen and stored at −20 °C until harvesting onto glass fiber filters. Incorporated radioactivity was determined on a MicroBeta Trilux 1450 scintillation counter (Wallac, Turku, Finland) and expressed as ccpm. Percentage (%) inhibition was determined by calculating 100 – ((ccpm ^3^H thymidin incorporation in the presence of antiserum/ccpm ^3^H thymidin incorporation in control cultures containing negative serum) × 100).

### 3.6. Animals

New Zealand White male and female rabbits weighing between 1.5 and 2 kg were purchased from Charles River Laboratories (Sulzfeld, Germany). Animals were kept in standard care facilities according to the guidelines of the Austrian Ministry of education, science and culture, and had free access to food and water (Ssniff^®^, Alleindiaet fuer Kaninchen, Ssniff Soest, Germany). The animal experiments had been approved and controlled by the municipal Veterinary Department of the City of Vienna (Austria).

### 3.7. Substances and Production of Antiserum

rTSST-1 wt and its mutant form rTSST-1 variant were produced in our laboratory. The rTSST-1 variant bears two mutations affecting the MHC binding site and the T-cell receptor binding site: G31R and H135A. The expression, purification and characterization of rTSST-1 wt and rTSST-1 variant were extensively described elsewhere [18]. All substances were tested for their chemical and biological properties in our laboratory and were proven to be below the detection limit of the Limulus test for LPS (0.01 EU/mL). Antisera were obtained in rabbits after four immunizations. Antiserum raised against wild-type TSST-1 had an ELISA titer of 46,330 and showed 99% inhibition of T-cell proliferation at a dilution of 1:100, when human PBMCs were stimulated with 0.3 ng/mL rTSST-1 wt. Antisera raised against TSST-1 variant had ELISA titers of 49,921 and 60,714 in two different rabbits and both sera showed 98% inhibition of T-cell proliferation when stimulated with 0.1 ng/mL rTSST-1 wt and an application of antiserum diluted 1:300.

### 3.8. Multiple Dose Lethal Challenge

Different quantities of TSST-1 wt (30 and 40 µg) in 1 mL sterile PBS were given subcutaneously twice a day for four days and once on day 5. Lethality was monitored over a period of 7–9 days.

### 3.9. Neutralization of rTSST-1 wt by Pre-Incubation with Antiserum

Thirty µg rTSST-1 wt were incubated in 1 mL undiluted antiserum at 37 °C overnight (neutralized toxin). Experimental design: two rabbits were challenged via multiple dose lethal challenge. Three rabbits were challenged with neutralized toxin at the same schedule. Three rabbits were challenged for three days (six injections) with toxin and, on days 4 and 5, with neutralized toxin.

### 3.10. Passive Immunization with Antiserum

One of two doses of rTSST-1 wt (30 µg; 40 µg) was dissolved in 1 mL PBS and given subcutaneously twice a day (at intervals of 6–8 h) for four days and once on day five. Two mL of antiserum was given subcutaneously either on days 2 and 3 or on days 3, 4, and 5 at different application sites. Lethality was monitored over a period of 7–9 days.

## 4. Conclusions

Taken together, our results demonstrate that treatment with neutralizing antibodies (hyperimmune serum) is protective late in the course of an ongoing exposure to TSST-1 in a multiple dose rabbit TSS model. In an *in vitro* system for monitoring the kinetics of cytokine release of human PBMCs after exposure to TSST-1 for defined periods, we show that IL-2 concentration in the PBMC cell supernatant increases up to 24 h after stimulation. Neutralization of the toxin at early as well as late time points during stimulation (e.g., at 7 h) leads to a significant reduction of IL-2 release in the supernatant. In contrast, TNFα secretion reached its maximum very early, e.g., at 5 h, and plateaued at this level up to 24 h. Therefore, neutralization of the toxin given after 5 h only had a marginal effect on the release of this cytokine.

It is well known that during T-cell activation, mRNA stability contributes significantly to changes in gene expression [35,36]. Large-scale gene expression profiling in activated T cells revealed that a large proportion of genes is regulated by mRNA stability [35,37]. Cytokine transcripts are labile [38] and regulated at the level of mRNA decay in a stimulus-specific manner. Activation of T cells by stimulation of CD28 led to selective stabilization of IL-2 and TNFα transcripts. In the absence of anti-CD28, mRNA degradation of these cytokines was rapidly induced [39]. We followed the kinetics of cytokine gene expression in the course of prolonged toxin exposure. The results of this study clearly indicate that in the presence of toxin, transcription of IL-2 and TNFα mRNA is ongoing and that the mRNAs of these cytokines are highly labile. It is likely that the gene expression of IL-2 and TNFα after exposure to TSST-1 is mainly regulated via mRNA turnover as it was shown for other modes of T-cell activation [39]. Both transcripts were rapidly induced upon stimulation with TSST-1. Neutralization of the toxin via addition of toxin-neutralizing antibodies immediately stopped cytokine gene transcription and the degradation of (already expressed) mRNA could be observed.

Interference with the prolonged stimulation of proinflammatory and immune activating cytokines, e.g., by inhibiting continual cytokine gene transcription, is likely to participate in the protective effect of neutralizing antibodies given as a postexposure treatment in animal models mimicking the clinical situation of prolonged toxin exposure such as observed in our multiple dose lethal rabbit model of TSS. At this stage, it is premature to present the mode of action of TSST-1-neutralizing antibodies in our animal model. Dissociation of superantigen bound to immune cells by neutralizing antibodies is one possible explanation whereby antibodies could protect late in the course of TSST-1 exposure. However, it is more likely that multiple activating hits over time, *i.e.* repeated activation of T cells by superantigenic toxins, rather than a single stimulatory event at the first encounter of immune cells with superantigenic toxins, leads to the prolonged hyperactivation of inflammatory responses characteristic of TSS. Using a TCR transgenic mouse model, it has been shown that prolonged antigen stimulation (thus delivering multiple activating hits over time) fully activates CD4 T-cell response [40]. Preliminary results indicate that in either case, high affinity antibodies produced after repeated immunization are required for superantigen neutralization as opposed to toxin-binding antibodies observed relatively early in the course of rabbit vaccination (data not shown). A candidate vaccine intended for use in humans has to be detoxified, e.g., by mutations in the toxin sites responsible for binding to TCR and MHC class II. Such a double mutant toxin might theoretically be less effective in inducing neutralizing antibodies as it lacks epitopes crucial for toxin activity. This was not the case in our system, as titers of neutralizing antibodies induced by double mutant and wild-type TSST-1 were comparable. This is in good agreement with previous studies showing that nonvirulent mutant toxins were immunogenic in animals and humans, e.g., a licensed acellular pertussis vaccine [41], a recombinant toxoid vaccine against *Clostridium difficile* [42], a recombinant *Clostridium perfringens* epsilon toxin mutant vaccine [43], and a mutant recombinant SEB vaccine [44]. Further studies are required to explore the mechanism(s) by which neutralizing antibodies administered late in the course of toxin exposure interfere with a prolonged toxin-mediated stimulation of immune cells. These studies should investigate important and currently unknown characteristics of the neutralizing antibodies involved, such as toxin-binding affinity, half-life of binding to TSST-1, and linear and conformational epitopes recognized by these antibodies.
